# Improved access to HIV diagnosis and linkage to antiretroviral therapy among children in Southern Nigeria: a before-after study

**DOI:** 10.1186/s12887-023-04050-w

**Published:** 2023-05-20

**Authors:** Esther Nwanja, Pius Nwaokoro, Uduak Akpan, Otoyo Toyo, Golda Ezeh, Iheanyichukwu Elechi, Helen Idiong, Titilope Badru, Olusola Sanwo, Augustine Idemudia, Satish Raj Pandey, Hadiza Khamofu, Moses Bateganya

**Affiliations:** 1 Achieving Health Nigeria Initiative (AHNi), Abuja, Nigeria; 2 FHI 360, Abuja, Nigeria; 3 FHI 360, Durham, NC USA

**Keywords:** HIV infection, Children, Orphaned, Case Identification, Linkage, Anti-Retroviral therapy, Nigeria

## Abstract

**Background:**

Globally, two out of five children living with HIV (CLHIV) are unaware of their HIV status, and a little more than 50% are receiving antiretroviral therapy (ART). This paper describes case-finding strategies and their contribution to identifying CLHIV and linking them to ART in Nigeria.

**Methods:**

This before-after study used program data abstracted during the implementation of different paediatric-focused strategies (provider-initiated testing and counselling, orphans and vulnerable children testing, family-based index testing, early infant diagnosis (EID), community-driven EID, and community-based testing) delivered in health facilities and in communities to improve HIV case identification. Data were abstracted for children (0 to 14 years) who received HIV testing services and were initiated on ART in Akwa Ibom State, Nigeria during the pre-implementation period (April–June 2021) and during the implementation period (July–September 2021). Descriptive statistics were used to describe the testing coverage, positivity rate (proportion of tests that were positive for HIV), linkage to ART, and ART coverage, by age, sex, and testing modality. Interrupted time series analysis (ITSA) on STATA 14 was used to estimate the effect of the implementation of these strategies on HIV testing uptake and positivity rate at a 0.05 significance level.

**Results:**

A total of 70,210 children were tested for HIV within the six-month period, and 1,012 CLHIV were identified. A total of 78% (n = 54,821) of the tests and 83.4% (n = 844) CLHIV were diagnosed during the implementation period. During implementation, the HIV positivity rate increased from 1.09% (168/15,389) to 1.54% (844/54,821), while linkage to ART increased from 99.4% (167/168) to 99.8% (842/844). The contribution from community-based modalities to CLHIV identified increased from 63% (106/168) to 84% (709/844) during the implementation, with the majority, 60.8% (431/709), from community-based index testing. Overall, ART coverage increased from 39.7 to 55.6% at the end of the intervention period.

**Conclusion:**

The findings show that expanding differentiated HIV testing approaches provided mostly in the community significantly increased pediatric case identification. However, ART coverage remains low, especially for younger age groups, and requires further efforts.

## Background

Globally, an estimated 2.8 million children and adolescents ages (0–19 years) are living with HIV, with only 54% receiving antiretroviral therapy (ART) at the end of 2020. About 310,000 new infections and 120,000 AIDS-related deaths were reported among children younger than 15 years in 2020 [1, 2]. Sub-Saharan Africa bears the brunt of HIV infection with 2.5 million children living with HIV (88% of the global burden), 260,000 new infections (89% of global infections) and 104,000 AIDS-related deaths [3].

Nigeria has the third-largest burden of HIV in children (190,000 children living with HIV) behind only South Africa and Mozambique [3]. Approximately 20,695 children aged 0–9 years were estimated to be newly infected with HIV in 2020, and about 30% of AIDS-related deaths occurred in CLHIV [4]. While treatment coverage in the country among adults increased from 65% to 2019 to 86% in 2020, coverage for children aged 0–14 years only increased from 36% to 45% within the same period [5, 6]. It is reported to be substantially lower in Akwa Ibom State, Nigeria [7, 8].

Different strategies have been used in isolation to improve treatment coverage among children [9–11]. The Paediatric ART Saturation Strategy (PASS) utilized targeted community-based testing to reach households of orphans and vulnerable children in Southern Nigeria [9]. In Cameroon, Penda et al. reported on the use of provider-initiated testing and counselling at multiple entry points within the facility to improve paediatric HIV case finding [10]. Yumo and colleagues recommended the use of targeted provider-initiated testing and counselling with symptom-based HIV diagnostic testing to improve HIV case finding among children in low-prevalence settings [11]. These isolated evidence-based strategies provide some improvements in the identification of HIV cases in children. However, it is necessary to implement a combination of approaches in program settings to achieve the maximum benefits of improving access to paediatric HIV care and treatment. Despite this, there is a limited understanding of the effect of a combination of strategies on improving access to HIV diagnosis and linkage in children. The United States Agency for International Development (USAID)-funded Meeting Targets and Maintaining Epidemic Control (EpiC) project in Nigeria implemented a mix of facility and community-based strategies to improve access to HIV diagnosis and treatment among children [12]. The purpose of this study is to examine the effect of a combination of facility and community-based strategies on access to HIV diagnosis and treatment for CLHIV in Nigeria. Specifically, the study sought to answer the following questions: What is the effect of combination of facility and community strategies on the number of children tested? What is the effect of the intervention on the number of new cases diagnosed and the number placed on antiretroviral therapy?

## Methods

### Study Design and Population

This was a before-after study conducted in 21 local government areas (LGAs) in Akwa Ibom State, Nigeria. We reviewed program data for children (0–14 years) who received HIV testing services between April and September 2021. The data were compared for two time periods: the pre-implementation period, from April to June 2021, and the implementation period, from July to September 2021. During the pre-implementation period, facility-based HIV testing services were largely used to identify CLHIV, while a mix of facility and community-based strategies was employed during the implementation period.

### Setting and program description

Akwa Ibom State, located in the southern part of Nigeria has an estimated population of 6,497,967 [13, 14]. Children aged 0–9 years make up 25% of the population, and those aged 10–19 years constitute another 24% [15]. The state has the highest HIV prevalence among adults (15–64 years) in Nigeria at 5.5% [16]. The HIV prevalence is 0.4% in children aged 0–9 years in the state, and 0.6% in those aged 10–14 years [17]. It is estimated that 10,064 children 0–14 years are living with HIV (2020 Spectrum Estimate). Access to HIV testing services within the State has been affected by the low HIV risk perception and the difficult geographic terrain, especially in the rural areas [8]. The resulting gaps in access to HIV services among pregnant women coupled with suboptimal antenatal attendance and delivery in health facilities have led to the high rate of mother-to-child transmission and a large burden among children [18, 19]. The EpiC project supported HIV care and treatment services in 21 LGAs—considered to have a high HIV burden—of the 31 in the states. Overall, 102 health facilities (1 tertiary, 29 secondary, and 72 primary health facilities) and 73 community ART management teams were supported [8].

### Description of the intervention

To improve treatment coverage among CLHIV, a paediatric task team was constituted to review and identify efficient HIV case-finding strategies. The team was composed of a consultant Paediatrician (as a clinical mentor), community team physicians (one per LGA), dedicated paediatric counsellor testers, and case managers. The team held key informant interviews with their caregivers of CLHIV on treatment and to understand contextual issues influencing the uptake of services. Brainstorming sessions were held with healthcare workers, PLHIV groups, civil society groups, community leaders, caregivers, and program staff to review program data and identify feasible models and evidence-based practices targeted at children that would be scalable within the operating environment [20]. Several strategies from existing literature were identified and reviewed for similarity and context. Six strategies were adopted, tailored to age-specific needs, and deployed at the facility and/or community levels during the implementation period. (Table 1).

Five of the six strategies were used in the pre-implementation period, but expanded in scope and reach with the addition of a sixth strategy (community-driven EID) during the implementation period. While the pre-implementation strategies were largely facility based with minimal community supplementation, the implementation strategies were largely community-based. For provider-initiated testing and counselling (PITC), healthcare providers offered to test children at risk of HIV who attend health facility clinics (such as immunization, outpatient, tuberculosis, and antenatal clinics). This was expanded during the implementation period into the community and offered at traditional birth attendant (TBA) homes and non-ART health facilities. For orphans and vulnerable children (OVC) testing, households enrolled on the OVC program were offered HIV testing and counselling in collaboration with other USAID partners. During family-based index testing (FBIT), biological children and siblings (younger than 15 years) of index HIV-positive clients were enumerated, and testing was extended into the community by dedicated testers who followed up with elicited contacts. Early infant diagnosis (EID) using dried blood spot (DBS) for HIV-exposed children younger than 18 months was scaled up, with testing conducted at a central laboratory. EID using DBS was expanded beyond the health facilities into community locations (community-driven EID) to cover the many out-of-facility births (up to 78%) occurring in these locations [19]. Point-of-care testing using GeneXpert® machines was introduced to complement the central laboratory testing in order to reduce the turnaround time for results and meet up the increased demand. The sixth strategy, i.e., community-based testing (CBT), testing was expanded by utilizing pediatric-focused testers in targeted hotspots, (such as at healing homes, patent medicine shops, creeks, and hard-to-reach areas), and using a combination of strategies (risk stratification, after-school testing [i.e., testing done when children were home from school], and holiday testing [i.e., testing done for children who were not available for testing during the school session]).

**Table 1:** Evidence-based strategies selected and implemented for finding children living with HIV in Akwa Ibom, Nigeria

Evidence-based testing strategies selected	Setting	< 1 year	1–4 years	5–9 years	10–14 years
Provider-initiated testing and counselling [21]	Facility and community	×	×	×	×
Orphans and vulnerable children testing [22]	Community only		×	×	×
Family-based index testing [23, 24]	Facility and community		×	×	×
Early infant diagnosis [EID] [23]	Facility only	×			
*Community-driven EID [24]	Community only	×			
Community-based testing [21]	Community only		×	×	×

* Strategy introduced during the implementation

Some testing strategies, such as FBIT, were used across multiple age groups, while others, such as EID, only applied to the youngest age group. Testing at the immunization clinics was focused on identifying missed opportunities among index children and their older siblings. Community ART management teams comprised of community mobilizers (for community entry and mapping), counsellor testers for testing, clinicians (for clinical management) and case managers (for case management and follow-up).

### Data Collection

Routine program data from patient records were reported daily into service registers and entered into the Lafiya Management Information System (LAMIS) [25] by trained data entry clerks and aggregate data was entered into the District Health Information Software 2 (DHIS2). All databases’ sources are validated weekly, monthly, and quarterly, following the national data reporting and validation guide. Different service registers were maintained for facility and community activities and data was reported into the relevant segments in DHIS2. For this analysis, program data were abstracted from LAMIS and DHIS2 for the pre-implementation period (April–June 2021) and implementation period (July–September 2021) respectively.

Data on the number of HIV tests conducted among children aged 0–14 years of age; and the number of positives identified were extracted from the DHIS2; while the information on children started on antiretroviral therapy, were extracted from the LAMIS. Quality checks were done to remove entries due to invalid, or duplicate data prior to analysis. Where there were missing data elements, such information was abstracted from the source document or patient care folders.

### Data analysis

Frequencies were used to summarize key outcomes across gender and other demographic characteristics. LGAs were categorized as urban, peri-urban, or rural.

The outcomes considered were case identification (number of HIV-positive tests), positivity rate (proportion of HIV tests that were positive), ART initiation (number of newly identified HIV-positive children initiated on ART) and linkage rate (proportion of newly identified HIV-positive children initiated on ART) for the pre-implementation and implementation periods. Children were considered HIV-positive if they had two positive nucleic acid tests on independent blood samples (in children less than 18 of age) and two positive antibody test results on independent blood samples in children greater than 18 months of age.

We conducted an interrupted time series analysis (ITSA) on STATA 14 to estimate the effect of the implementation of these strategies on HIV testing uptake and positivity rate. The model, however, was fitted to weekly level data to account for variations that occurred with the change in strategy implementation. T-test statistic was conducted to compare the difference in average testing within the two periods.

Kaplan Meier’s log-rank test was used to compare time to ART for both periods. Also, ART coverage for both periods was compared using the Mann-Whitney U test with a 0.05 significance level. The baseline for ART coverage was computed from the HIV spectrum estimate data of the CLHIV burden of the 21 supported LGAs.

## Results

A total of 70,210 children were tested for HIV tests over six months: 15,389 were tested during the pre-implementation period and 168 CLHIV identified, 54,821 were tested during the implementation period and 844 CLHIV identified (Table 2). Of the 844 HIV-positive children identified during the implementation, 3.7% were children aged less than one year, 23.8% were 1–4 years, 25.9% were 5–9 years and 46.6% were 10–14 years. Of the 813 HIV-positive children aged 1–14 years, 50.3% (409) were males. (Table 2).

**Table 2:** HIV Testing and case identification rates pre-implementation and during the implementation period by age groups from April 2021 to September 2021, Akwa Ibom, Nigeria

		Pre-implementation				Total	Implementation				Total	p-value
		< 1 year	1–4 years	5–9 years	10–14 years		< 1 year	1–4 years	5–9 years	10–14 years		
Overall												
	CTRR	273	4696	6118	4302	15,389	534	12,167	21,806	20,314	54,821	< 0.001
	Positive	16	55	38	59	168	31	201	219	393	844	< 0.001
	Yield	5.90%	1.20%	0.60%	1.40%	1.10%	5.80%	1.70%	1.00%	1.90%	1.50%	<0.001
Sex*												
Male	CTRR		2248	3014	1918	7180		5984	10,497	9531	26,012	< 0.001
	Positive		22	16	20	58		112	122	175	409	< 0.001
	Yield		1.00%	0.50%	1.00%	1.00%		1.90%	1.20%	1.80%	1.60%	< 0.001
Female	CTRR		2448	3105	2396	7949		6183	11,309	10,783	28,275	< 0.001
	Positive		33	22	39	94		89	97	218	404	< 0.001
	Yield		1.40%	0.70%	1.60%	1.20%		1.44%	0.90%	2.00%	1.40%	<0.001
Testing modality												
Facility	CTRR	273	2106	1901	1486	5766	534	4176	4786	3801	13,297	0.001
	Positive	16	22	12	12	62	31	29	25	51	136	0.001
	Yield	5.90%	1.00%	0.60%	0.80%	1.10%	5.80%	0.70%	0.50%	1.30%	1.00%	< 0.001
Community	CTRR		2590	4218	2814	9622		7991	17,020	16,511	41,524	< 0.001
	Positive		33	26	47	106		172	194	342	708	< 0.001
	Yield		1.30%	0.60%	1.70%	1.10%		2.20%	1.10%	2.10%	1.70%	0.174
Settings												
Urban	CTRR	145	891	960	961	2957	217	2511	4014	6211	12,953	< 0.001
	Positive	3	11	6	13	33	6	17	17	62	102	0.002
	Yield	2.10%	1.20%	0.60%	1.40%	1.10%	2.80%	0.70%	0.40%	1.00%	0.80%	0.964
Peri-urban	CTRR	57	1894	2739	1544	6234	135	5155	10,011	7799	23,100	< 0.001
	Positive	5	15	12	16	48	7	83	86	194	370	< 0.001
	Yield	8.77%	0.80%	0.40%	1.00%	0.80%	5.20%	1.60%	0.90%	2.50%	1.60%	< 0.001
Rural	CTRR	71	1911	2419	1797	6198	182	4501	7781	6304	18,768	< 0.001
	Positive	8	29	20	30	87	18	101	116	137	372	< 0.001
	Yield	11.30%	1.50%	0.80%	1.70%	1.40%	9.90%	2.20%	1.50%	2.20%	2.00%	< 0.001

*Data for EID done for children < 1 year was not documented by facility and community setting

CTRR- Counselled tested and received results

**Fig. 1:**
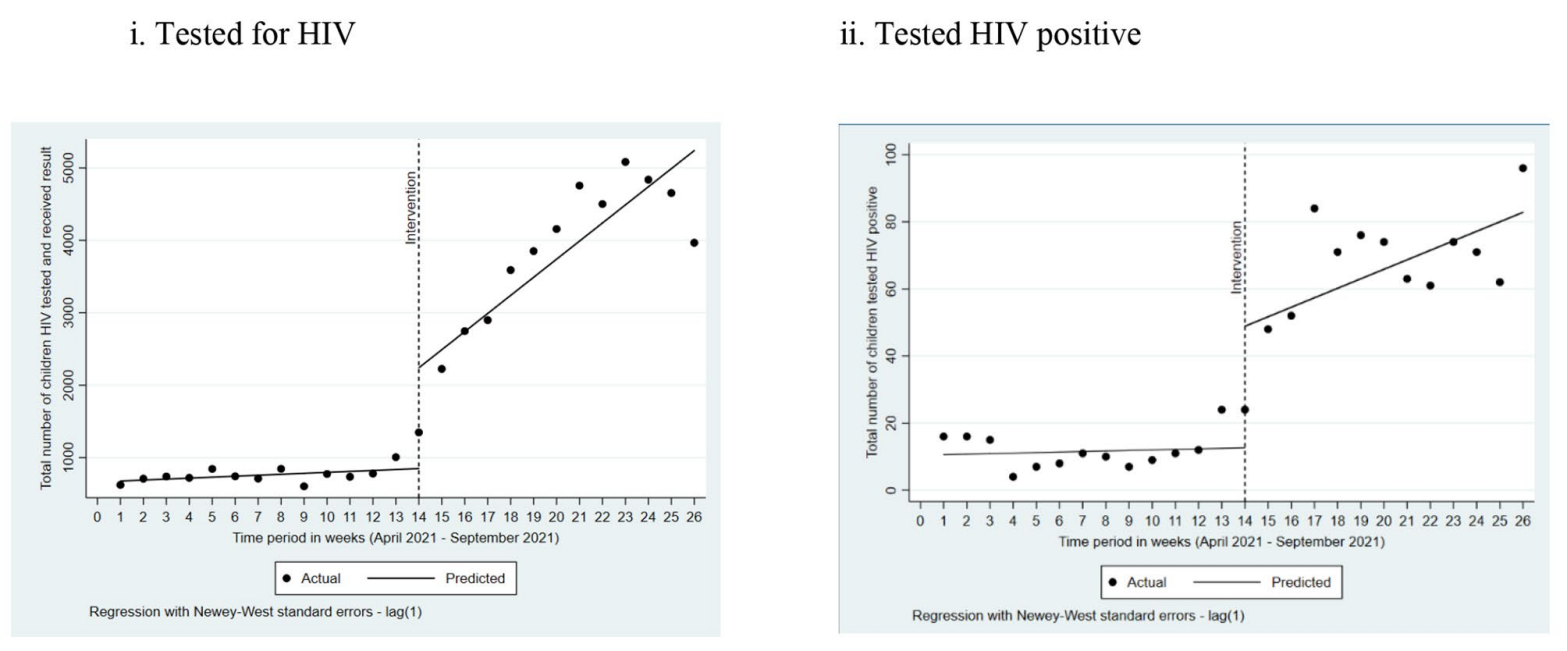
Actual and predicted trends in the total number of children (i) tested for HIV and (ii) tested HIV positive, using ITSA, by weeks (April–September 2021) in Akwa Ibom, Nigeria

There was no significant improvement in the weekly number of children tested for HIV pre-implementation (β = 13.26; p = 0.069; CI= [-1.11, 27.6]) (Fig. 1[i]). The number of children tested for HIV and received test results increased by 1,390 (β = 1,390.6; p = 0.002, CI= [560.9, 2220.3]) in the first week of intervention and was followed by significant weekly increases in HIV tests conducted (relative to the pre-implementation phase) (β = 236.8; p = 0.001, CI= [104.72, 368.79]) (Fig. 1[i]).

Similar to testing, there was no significant improvement in HIV-positive cases identified every week pre-implementation (β = 0.16; p = 0.778; CI= [-0.996, 1.314]) (Fig. 1[ii]). However, the number of HIV-positive children identified increased by 36 (β = 36.2; p = 0.008, CI= [10.32, 62.13]) in the first week of the intervention, followed by significant weekly increases in the number of positive children identified (relative to the pre-implementation phase) (β = 2.67; p = 0.042, CI= [0.10, 5.24]) (Fig. 1[ii]).

The positivity rate increased from 1.1% (168/15,389) to 1.5% (844/54,821) between the two periods. This increase was seen across all age groups except among children less than one year where the positivity rate marginally reduced from 5.9 to 5.8%.

The relative increase in the absolute number of children tested and those diagnosed with HIV across both periods was higher among males (3.6 times and 6.2 times, respectively) and in the community setting (4.3 times and 6.6 times, respectively) (Table 2). Compared to pre-implementation, testing rates were higher among older children (3.6 times) compared to infants (2.0 times). Similarly, HIV case identification was higher among older children (5.3 times) compared to infants (1.9 times). However, while the number of children tested was noticeably higher in urban LGAs (3.7 times), more HIV-positive children were identified in peri-urban LGAs (7.7 times).

Community-based testing had the highest contribution to HIV testing during the implementation period (n = 27,460 50.1%), while community index testing had the highest contribution to CLHIV identified (n = 431, 51.07%) (Table 3).

**Table 3:** Contribution of strategies used during the implementation period to the number of children tested, CLHIV identified, and HIV positivity yield, Akwa Ibom, Nigeria (July-September 2021)

Targeted testing strategies	Tested and received HIV result			Identified CLHIV		Positivity rate
		(n = 54,821)	% Contribution to case finding	(n = 844)	% Contribution to case identification	
**Provider-initiated testing and counselling (PITC)**						
Facility PITC		5250	9.6%	22	2.6%	0.4%
Community PITC		38	0.1%	1	0.1%	2.6%
**Family-based index testing**						
Facility index testing		4276	7.8%	75	8.9%	1.8%
Community index testing		14,035	25.6%	431	51.1%	3.1%
**Orphans and vulnerable children testing**						
Testing for orphans and vulnerable children		3228	5.9%	7	0.8%	0.2%
**Community-based testing**						
Community-based testing		27,460	50.1%	277	32.8%	1.0%
**Early infant diagnosis testing**						
Early infant diagnosis testing		534	1.0%	31	3.7%	5.8%

CLHIV _ Children Living with HIV

**Fig. 2:**
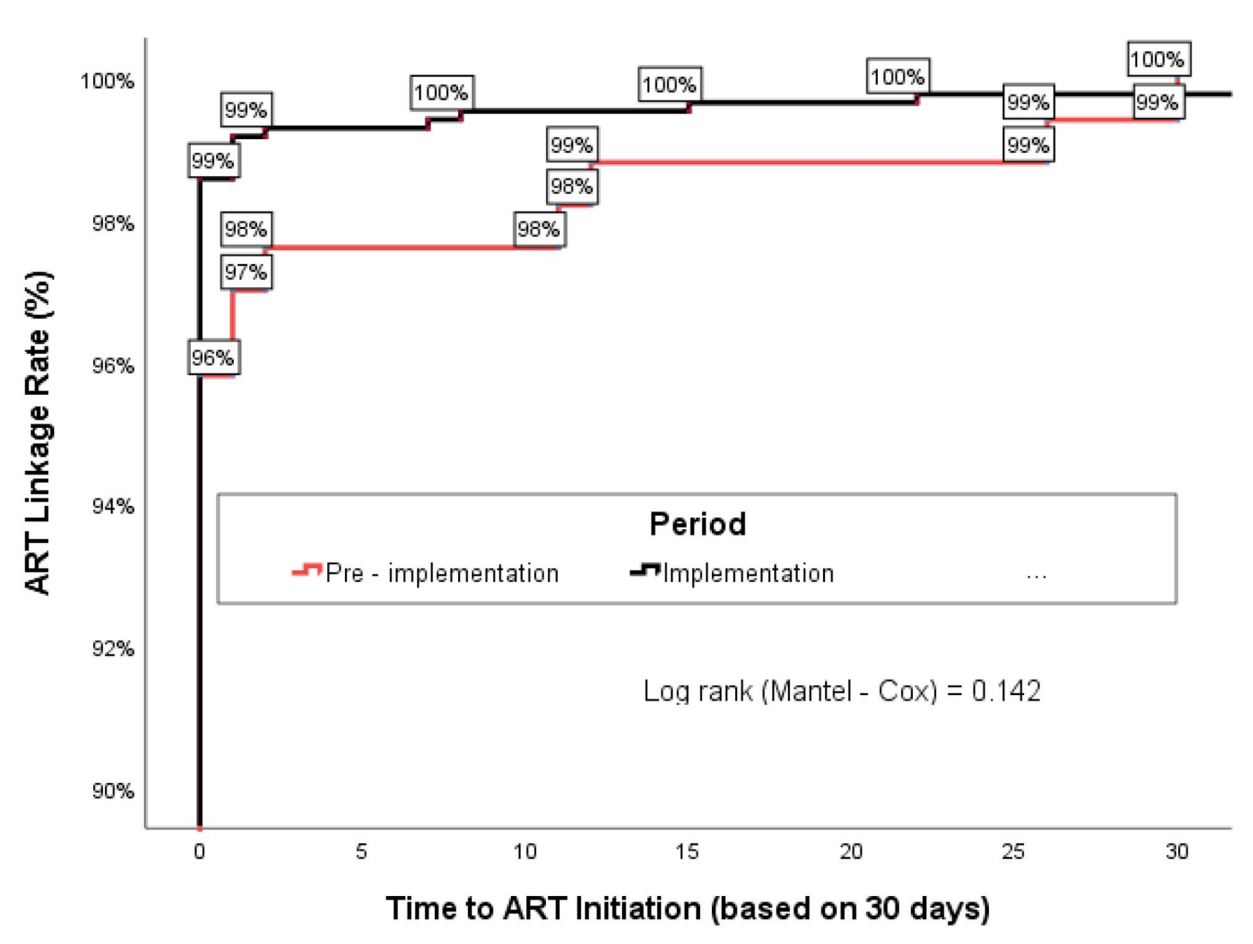
Time from diagnosis to ART initiation among the CLHIV started on ART during the pre-implementation and implementation periods, Akwa Ibom, Nigeria (April–September 2021)

Same-day linkage to ART increased from 95.8% during the pre-implementation phase to 98.6% during the implementation phase. Similarly, the cumulative linkage rate, calculated at the end of 30 days, increased marginally from 99.4% (167/168) in the pre-implementation period to 99.8% (842/844) during the implementation period (p = 0.142) (Fig. 2).

At the end of the implementation period, ART coverage was 55.62% (Table 4) overall. By age group, ART coverage was 10% (67/652), 48% (1,069/2,242), 63% (1,409/2,223), and 69% (1,518/2,188) for CLHIV ages < 1, 1–4, 5–9 and 10–14 years, respectively. There was a relative increase in the number of CLHIV identified and linked to ART across all age bands.

**Table 4:** Pre-implementation and implementation period ART coverage by age group, Akwa Ibom State, April 2021 – September 2021

Age Group	Pre-intervention					Intervention		
	CLHIV Estimate	Achievement	% Achieved			Achievement	% Achieved	
**< 1**	652	22	3%			67	10%	
**1–4**	2,242	747	33%			1,069	48%	
**5–9**	2,223	1,113	50%			1,409	63%	
**10–14**	2,188	1,015	46%			1,518	69%	
**Total**	7,305	2,897	39.7%			4,063	55.6%	

## Discussion

The results of this analysis show a 3.6-fold increase in the number of children 0–14 tested, a 5.2-fold increase in HIV-positive children identified, and a 98.6% same-day ART initiation following the implementation of a multi-pronged strategy. Targeted community-based HTS, facility and community-driven EID, testing of orphan and vulnerable children, Family-based index testing, and PITC contributed to high rates of case identification. Overall, the treatment coverage for CLHIV improved from 39.7 to 55.6% within the period, highlighting progress but also the need for more intensified HIV case finding to identify undiagnosed children.

Different testing strategies used singly or in combination, improve HIV case finding in children [26–29]. The results of this programmatic intervention that combined facility and community-based strategies were consistent with findings from other HIV programs [30–32]. While facility-based intervention targeted those attending facilities for other needs, community-based interventions centred around CAM teams and leveraged local health structures such as patent medicine vendors, traditional birth attendants, and healing homes to reach previously undiagnosed children. The Nigeria National Task Shifting Policy promoting lay workers’ use was a critical enabler [33].

The positivity rate (yield) from community HIV testing was higher in this study (1.7%) than in other settings where community testing has been conducted [28]. Using multiple strategies adapted to the local context, and the additional use of risk stratification tool to identify children more likely to be HIV positive, likely contributed to the high positivity rates. Improved tested efficiency among children using screening tool has been reported by Antelman and colleagues [34]. The addition of risk screening to community testing is especially important in countries like Nigeria where the prevalence among children is low to ensure more efficient use of meagre resources [17, 35].

Index testing is a well-recognized strategy for efficient HIV case finding, especially in low-prevalence settings and subpopulations [36, 37]. In the Akwa Ibom setting, it was implemented both at the facility and community levels. Community index testing resulted in a high yield of 3.1% and contributed 51.0% of all HIV-positive children identified. The case-finding rate from this modality is higher than that reported in Lesotho (0.7%), where door-to-door (D2D) index testing was used to complement facility-based index testing [26]. The difference may be because Lesotho was closer to achieving ART saturation among children compared to our setting where the ART saturation among children was lower [38].

Although significant improvements in case finding were seen in all age groups, the largest proportion (47%) of CLHIV identified were older children (10–14 years). Similar studies from Kenya and Zimbabwe identified slightly younger children, with median ages of 8 and 11 years, respectively [39–42]. This could suggest missed opportunities for early HIV diagnosis among antenatally or perinatally infected children, or new infections among adolescents. Integration with recency testing could help provide more useful information.

The lack of timely EID has been documented as a reason for poor uptake of treatment among HIV-exposed infants [43]. Extending EID services to the community was novel and increased the uptake of testing by 84%. Late infant testing is associated with poor antenatal care (ANC) attendance and is especially common in parts of Nigeria where there is high patronage of traditional birthplaces among pregnant women [19, 44]. The general lack of adherence to standard precautions of infection control, as well as precautions to prevent vertical transmission of HIV from mother to child in these traditional settings, lack of education about safe breastfeeding options, and lack of skilled birth attendants, could be responsible for the high proportion of children who tested positive (5.8%) with this strategy.

The results showed that linkage to ART was optimal across all age groups, unlike in Lesotho, where Sindelar, K. and colleagues reported a linkage rate of 82% after 12 months from community-based interventions [26]. In our setting, the linkage gap was closed using the community ART management team [8]. Each mobile community team had physicians and case managers who were trained in providing pediatric-focused care. Once CLHIV were identified, they were immediately seen by a physician who conducted an initial clinical evaluation and ascertained their readiness to commence treatment. CLHIV who were deemed to have good psychosocial support were initiated on treatment, while those who were not, were deferred and linked to other services.

The study had some limitations. The main weakness of before and after designs is the lack of a control group. This limits the value of information obtained on the intervention outcome link. Without a control group, it is difficult to establish the cause-and-effect relationship between the intervention and the outcome and to account for possible confounders. Furthermore, the analysis covered three months and did not account for how these strategies would affect results in the long term, including the cost-effectiveness of these strategies. However, these interventions have been implemented beyond the study period. Lastly, HIV testing data for EID services were documented at the facility level for both community- and facility-driven EID efforts; thus, it was difficult to attribute contributions from the community and facility. However, the difference between periods in the total number of samples collected emphasizes the value of community-driven EID.

Despite these limitations, this study is the first community-wide intervention that used multiple testing strategies in Nigeria, thus, providing insights to policymakers and program implementers on the use of differentiated HIV testing for case identification among children. As one of the countries with the highest burden of HIV, scaling up these interventions could help Nigeria end AIDS in children by 2030. The results from differentiated testing strategies provides evidence for managers, policymakers, and other stakeholders to inform decision-making and improve implementation of HIV testing programs from implementation design to resource allocation.

## Conclusion

The study showed that the combination of multiple strategies in facility and community setting rapidly improved access to HIV diagnosis among children. Although the overall yield from HIV testing for children is low and requires an enormous amount of resources, index testing remained the most efficient strategy for HIV case finding. Treatment programs designed for children living with HIV should incorporate community-based services to facility-based services to achieve rapid results. More strategies can be explored to reach this younger age group.

## Data Availability

The datasets used and/or analyzed during the current study are available from the corresponding author on reasonable request.

## Abbreviations

ANC: Antenatal care
ART: Antiretroviral therapy
CBT: Community-based testing
CLHIV: Children living with HIV
DHIS2: District Health Information Software 2
EID: Early infant diagnosis
EpiC: Meeting Targets and Maintaining Epidemic Control
FBIT: Family-based index testing
HTS: HIV testing services
ITSA: Interrupted time series analysis
LGA: Local government area
OVCT: Orphans and vulnerable children testing
PCR: Polymerase chain reaction
PEPFAR: U.S. President’s Emergency Plan for AIDS Relief
PITC: Provider-initiated testing and counselling
PMTCT: Prevention of mother-to-child transmission of HIV/AIDS
SIDHAS: Strengthening Integrated Delivery of HIV/AIDS Services
TBA: Traditional birth attendant
USAID: United States Agency for International Development
